# Piezo1 ion channel: a core target for mechanotransduction in orthodontic alveolar bone remodeling

**DOI:** 10.3389/fcell.2026.1800626

**Published:** 2026-05-08

**Authors:** Yan Yan, Xian-Zhuo Chen, Peng Na, Shi-Han Gan, Jing-Lun Jiang, Lan Wang, Rui Zuo, Li-Hua Li

**Affiliations:** 1 Department of Stomatology, Affiliated Hospital of North Sichuan Medical College, Nanchong, China; 2 Biotechnology Innovation Drug Application and Transformation Key Laboratory of Sichuan Province, North Sichuan Medical College, Nanchong, Sichuan, China; 3 Department of Burn and Plastic Surgery, Affiliated Hospital of North Sichuan Medical College, Nanchong, China

**Keywords:** Piezo1 channel, orthodontic tooth movement, mechanotransduction, bone remodeling, osteoblasts, osteoclasts

## Abstract

Physiological tooth movement during the process of orofacial rehabilitation, as well as the degree of skeletal responses to orthodontics procedures, depends on the bone remodeling processes that are controlled by the mechanosensitive receptors. Piezo1 has become one of the receptors of great attention over the last few years. Besides acting as a sensor of the mechanical microenvironment, Piezo1 is also important in alveolar bone remodeling because of its ability to regulate osteocyte activity. This is a review of how Piezo1 mediates orthodontic tooth movement (OTM) by detecting and transducing mechanical stimulating factors using various molecular pathways and by regulating the activities of osteoblasts and osteoclasts. Most recent research developments are summarized with the aim of determining the impact of Piezo1 channel inhibition on orthodontic tooth movement as well as elucidating the molecular pathways involved in the same. Additionally, the prospective clinical use of Piezo1 as a therapeutic target of orthodontic therapy is discussed. We also highlight the importance of Piezo1 in regulating bone formation and maintaining the balance between osteogenesis and osteoclastogenesis. In general, the results can be used to optimize the method of providing orthodontic treatment and develop new strategies in the field of orthodontics.

## 1 Introduction

Orthodontic tooth movement (OTM) is a complex biomechanical process, and the physical load imposed on the teeth triggers complex biological cascade reactions within the Tooth Week tissue. This shift is largely dependent on the adaptive remodelling of the toothbone, which is managed by a delicate synergy between the skeletal and broken cells. The core of the biological response lies in the ability to maintain and transform cytological sensory mechanics into biochemical signal -- a phenomenon known as mechanical transfer.

In recent years, the Piezo1 ion channel has emerged as a cornerstone of mechanosensing in various tissues, specifically within the dental environment. In the course of the treatment, mechanical stress activated the Piezo1 channel within the PDL, immediately triggering the ca^2+^ion internal flow outside the cell. Such changes in intracellular ion concentrations change the expression of critical bone conversion genes (Luu et al., 2024). Specifically, there is a critical balance between Piezo1 signal-routing and RANKL (receptor activator of nuclear factor kappa-B ligand). RANKL is a crucial factor in osteoclastogenesis, whereas osteoprotegerin (OPG) exerts inhibitory effects on osteoclast activity (Ilka et al., 2022).

The theoretical basis for this review is derived from the rapid accumulation of evidence of multiple effects of Piezo1 in dental membrane (PDL) cells and bone remodelling. As we move towards a more individualized and biologically driven strategy, there is an urgent need to integrate current research on the impact of Piezo1 on OTM. Therefore, this paper is intended to provide an overview of how Piezo1 is able to induce the movement of orthodontically-transformed teeth through multiple molecular route detection and transfer of mechanical factors. We have further summarized recent research progress to determine the impact of Piezo1 inhibition on OTM, to clarify its potential molecular network and to explore the prospects for its clinical application as a target for treatment in advancing orthotic treatment.

To conclude, Piezo1 is not only involved in the orthodontic movement of teeth, but it is also strongly related to the mechanisms of mechanotransduction and the accompanying bone remodeling signals and inflammatory pathways triggered by the forces (Dong et al., 2025). More studies are needed to understand the electric sensitivity of Piezo1 in various mechanical loading conditions and also to explain the mechanisms. Moreover, it is hoped that the future clinical research will expand the therapeutic capabilities of Piezo1 in orthodontics, thus enhancing the safety and efficacy of orthodontic treatments (Du and Yang, 2023).

## 2 Main body

### 2.1 Mechanism of mechanical sensing and signal transduction in the Piezo1 channel (Figure 1)

#### 2.1.1 Structural features and mechanical sensitivity of Piezo1

Piezo1 is a mechanically sensitive ion channel, and its unique subunit structure enables it to respond to extracellular mechanical stimuli. Piezo1 is a homotrimer, comprising three identical subunits, which can form a large trimeric structure with an ion-conducting channel in the center. When activated, cellular substances such as Ca^2+^among others are thought to diffuse across the cell membrane (Hao et al., 2024). Also, it is reported that Piezo1 activity and inactivity is tightly linked with mechanical thresholds (Wong et al., 2021). Orthodontic force exerts mechanical stretch and pressure on the cell membrane. These forces trigger conformational changes in Piezo1, leading to the opening of the ion channel. The resulting pore activation facilitates a significant influx of Ca 2+ into the cytoplasm. As a result, the activation affects downstream targets and pathways of signaling related to bone remodeling (Lai et al., 2025).

**FIGURE 1:**
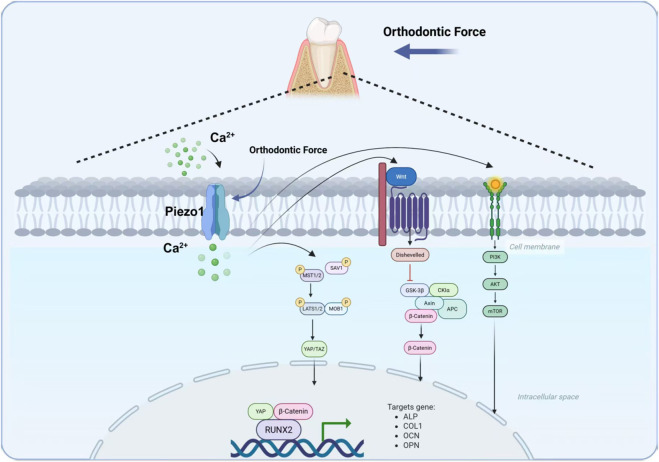
This figure illustrates the molecular mechanism by which the mechanosensitive ion channel Piezo1 regulates alveolar bone remodeling under orthodontic force through multiple signaling pathways, achieving the molecular network of adaptive alveolar bone remodeling under orthodontic force via multi-pathway interactive regulation of osteogenesis-related gene expression.

#### 2.1.2 Transduction downstream signalling pathways of mechanical stimulus

An increase in intracellular free calcium caused by Piezo1 stimulation has major effects on mitochondrial physiology (He et al., 2024). Moreover, this influx of calcium triggers a number of other signal pathways, such as the YAP/TAZ. This is a pathway that is playing a key role in cell proliferation and cell survival and it is mainly defined by transcriptional coactivators YAP (Yes-associated protein) and TAZ (transcriptional coactivator with PDZ-binding motif). Piezo1 stimulation causes the Ca^2+^ influx into the cell, which in turn triggers their nuclear translocation, which functions as a mechanoregulator of YAP/TAZ. This translocation and successive amplification of their regulatory power have been connected to osteogenesis and cell repair (Jurynec et al., 2024; Yang et al., 2024). Moreover, in Piezo1-mediated activities the processes of mitochondrial dynamics and intercellular signaling which play a critical role in the growth and development of teeth have been impacted (Lin et al., 2022).

#### 2.1.3 Piezo1 spatiotemporal expression properties with orthodontic forces

The role of piezo1 is the essential condition of apoptosis of PCO cells, which is carried out by orthopedic force. In line with its role in bone remodelling and tooth movement, moderate pressure induces Piezo1 expression (Ru et al., 2024), which varies over the day and on the levels of its expression. Piezo1 is maximally induced directly after initial pressure application at specific locations. It is worth noting that compression and tension regions are highly expressed compared to strain regions, which differs with the previous reported Piezo1 expression patterns (Seidel et al., 2023). This difference is likely to result from differences in the oral tissue mechanical load programme. As the main mechanical sensor, Piezo1 is capable of effectively transforming the external positive load into a series of cytological cascades. Structural adaptation and functional transformation observed in these high-resilient regions further confirm the central role of the tunnel in driving the organization to reshape. These results show that Piezo1 activity is essential for achieving efficiency OTM (Radermacher et al., 2024).

### 2.2 Interaction between Piezo1 and bone cell activity ((Figure 2))

#### 2.2.1 Mechanism of Piezo1 regulation in osteoblast differentiation

Piezo1 is a key mechanosensitive ion channel that is involved with cell electrical activity. Recent research findings have revealed that the Piezo1 controls the expression of RUNX2, which is a critical osteogenesis transcription factor. RUNX2 expression in osteoblasts, which are involved in differentiation of osteoclasts and formation of tooth structure, is enhanced after exposure to mechanical strain or Piezo1 agonist Yoda1 (Guerrero et al., 2020). Yoda1 stimulates PI3K/Akt pathway and increases magnesium level in the cell. In contrast, Piezo1 inhibition is also a serious issue in the expression and activity of RUNX2, which is a key protein in bone differentiation (Sasaki et al., 2020; Zeng et al., 2022).

**FIGURE 2:**
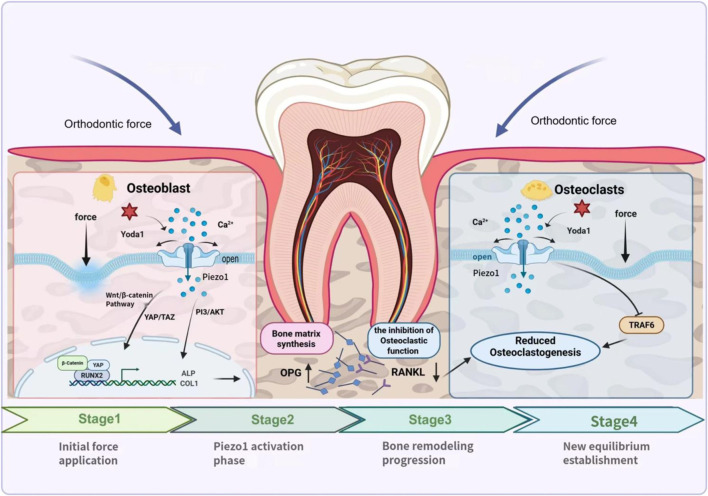
The figure illustrates the four-stage mechanism by which the Piezo1 mechanosensitive ion channel, under orthodontic force, regulates the signaling pathways of osteoblast-mediated bone.

There are a number of osteoblastic markers that are expressed in cells during bone formation such as alkaline phosphatase (ALP) and type I collagen (COL1). Piezo1 expression is upregulated during bone formation and mineralization, thereby promoting the expression of ALP and COL1 mRNA (Kim et al., 2023). This is strongly associated with the process of regulating intracellular potassium ions. Local spine formation occurs via mechanical stimulation, which raises calcium influx via Piezo1 channels provoking downstream signaling cascades (Francisco et al., 2020). The osteogenic factors are produced in response to the interactions of the osteoclast-associated mechanosensor Piezo1, which further promotes bone formation.

Piezo1 is, therefore, one of the main mechanosensors of bone formation, and mechanical stimulation is one of the most crucial regulatory inputs. Activation of Piezo1 channels through physical stimulation causes the rapid increase in intracellular Ca ^2+^ levels, which promotes PI3K-Akt and YAP pathways. The routes enhance bone matrix formation and mineralization, which stimulate bone growth and differentiation (Ochiai et al., 2024; Wang C. et al., 2023). Furthermore, Piezo1 has a wide range of interactions with epithelial tissues, indicating that its signaling is involved in the immunomodulatory regulation of bone metabolism (Zhou et al., 2025). Taken together, these results enhance our current knowledge of Piezo1-regulated osteogenesis and furnish an experimental foundation on the design of a specific approach in bone repair.

#### 2.2.2 Piezo1’s rules in osteoclast creation and functionality

Piezo1 is required in the formation and functional activity of bone precursor cells. It has been shown that there is a great correlation between the functional capacity of these cells and the level of expression of the Piezo1 ion channel (Murakami et al., 2025). Piezo1 activation regulates the growth of bone precursors and their differentiation as well as down-stream signals that have a strong connection with bone remodeling, especially when they experience mechanical loading. Piezo1 activation through this pathway regulates bone resorption by facilitating a quicker response of precursor cells to the signal with structural changes (Lin et al., 2025; Uchinuma et al., 2024).

The primary factors in osteoclast differentiation are osteoprotegerin (OPG) and receptor activator of nuclear factor OPG and RANKL, and the ratio of their expression in bone cells is the most actively discussed issue lately. Nonetheless, the actual impact of Piezo1 on OPG remains unclear. Through some experimental circumstances, OPG protein and mRNA production is elevated (Tariq et al., 2022). Conversely,RNA interference, GsMTx-4, a mechanosensitive channel inhibitor, or Piezo1 knockout lead to higher levels of RANKL production and lower levels of OPG (Uchinuma et al., 2024).

Piezo1 ion channels promote bone resorption by directly inhibiting the Piezo1 ion channel network with mechanical stimulation. The osteoclasts control their intracellular magnesium concentration by detecting mechanical signals through Piezo1 and allowing magnesium ions to enter the cell (Vasileva et al., 2024). Inhibition of NFATc1 expression during osteoclastogenesis via this signaling axis exerts a negative regulatory effect on excessive bone resorption. Thus, Piezo1 plays a central role not only in normal bone homeostasis but also in the regulation of bone-related mediators, highlighting its critical role in maintaining the balance between osteoblasts and osteoclasts (Wang et al., 2025).

#### 2.2.3 Piezo1-mediated crosstalk between osteoblasts and osteoclasts

One of the mediators of the bidirectional communication between osteoblasts and osteoclasts is Piezo1. Mechanical information is transduced by Piezo1 channels in osteoblasts which then release osteogenic and regulatory factors that regulate osteoclast differentiation and resorptive activity through paracrine signaling pathways (Ni et al., 2025). Activation of Piezo1 in osteoblasts also stimulates the secretion of osteoprotegerin (OPG), a decoy receptor of RANKL and thus suppressing the development of osteoclastic precursors in the presence of mechanical loading, thus maintaining bone homeostasis (Zhang et al., 2024).

Piezo1, in bone remodeling, therefore, acts as both a mechanosensor and intercellular signaling mediator by integrating the differentiation of osteoclastogenesis and osteoclast resorptive activity by regulating the release and production of key coupling factors like RANKL and OPG (Yang et al., 2025).

This mechanically sensitive ion channel is fundamental to maintaining bone density and structural mass. It achieves this function by accurately coordinating the collection and functioning of bone formation and bone absorption cells (Du et al., 2024). Empirical data indicate that Piezo1 activation has a dual modularization effect: it promotes bone cell separation while inhibiting bone cell formation. This synchronous regulation mechanism is the cornerstone for maintaining the structural integrity of the tooth cell bones under mechanical loads (Hu et al., 2023). This mutual control clarifies our knowledge of bone metabolic regulation and indicates that Piezo1 is a promising treatment option in the prevention and therapy of bone disease.

### 2.3 Effects of Piezo1 inhibition on tooth movement

#### 2.3.1 Effects of Piezo1 inhibition in vitro experiments

Piezo1 pharmacological inhibition or silencing of Piezo1 silencing using siRNA has a significant effect on osteoblast activity and bone remodelling *in vitro* (Zhou et al., 2025). Mechanical stresses on periodontal ligament fibroblasts (PDLFs) which are then treated with GsMTx4, a specific Piezo 1 inhibitor demonstrate a decrease in RANKL expression and osteoblastic differentiation (Du et al., 2024). These findings further confirm the central role of Piezo1 in the mechanical transfer response of skeletal cells. In addition, the internal biopsy model shows that the absence of Piezo1 leads to significant reorganisation of the cortical bone structure. The fact that mice lacking the route showed a serious imbalance between bone formation and broken bones highlights the indispensable role of Piezo1 in managing both bone formation and bone absorption (Ochiai et al., 2024).

Mechanosensitive ion channels such as the Piezo1 are also involved in mechanical stimulation which prevents overreacting of the cells to external mechanical stimuli. Mechanically induced intracellular Ca ^2+^influx in PDL fibroblasts is inhibited by silencing of Piezo1 and accompanied by downregulation of major intracellular signalling pathways (Dong et al., 2025). Therefore, it is possible to conclude that Piezo1 is not only a major mechanosensor in cells of the bone area but also a significant regulator of endogenous biochemical processes. Piezo1 activity regulation can also potentially affect the alkaline phosphatase (ALP) levels in PDL fibroblasts, based on *in vitro* results (Wang et al., 2025). Piezo1 stimulation enhances the responsiveness of PDL fibroblasts to mechanical stress, induces the secretion of proinflammatory cytokines and enhances the functional capacity of PDLFs to mediate OTM (Schröder et al., 2020). Taken together, these data demonstrate that Piezo1 is involved in osteocyte bioactivity as well as in the intricate transduction of mechanical signals that are the basis of tooth movement and bone resorption.

#### 2.3.2 Piezo1-mediated regulation of tooth movement in animal models

Experimental evidence *in vivo* indicated that Piezo1 inhibition significantly affects the scale and speed of orthodontic movement of teeth. Piezo1 direct blockade with the use of GsMTx4 or PWM causes a substantial decrease in the distance of tooth movement, which is in agreement with the established role of Piezo1 in periodontal tissues (Wang et al., 2024). On the other hand, Piezo1 stimulation promotes periodontal tissue remodeling during orthodontic force, and inhibition suppresses tooth movement, which needs to be acknowledged as an invaluable contribution of Piezo1 to force-induced movements in the tooth (Lin et al., 2022). The quantitative analysis also shows that Piezo1 blockade is not only able to reduce the overall distance of tooth movement, but also to modify its time-varied kinetics. Profiling of movement rates with the help of the previously published data showed that the velocity of the tooth movement decreased progressively over time in the Piezo1-inhibited groups, in comparison to the controls (Dong et al., 2025). Additionally, the experiments on the animals show that Piezo1 inhibition impairs the microarchitecture of the alveolar bone, which results in inadequate bone remodeling, and, consequently, the stability and health of the alveolar bone is compromised (Lipani et al., 2025).

The abnormal alterations in the osteoblast and osteoclasts populations in the alveolar bone are alleviated when Piezo1 is pharmacologically acted upon and osteoclast activity is suppressed especially (Jiang et al., 2021; Zhu et al., 2021). Histological and microscopic examinations show a decrease in the number of osteoblasts and weakening of the osteoclastic activity in alveolar bone after treatment with Piezo1 inhibitors. Such a reduction in bone remodeling capacity correlates with the reduced ability to regenerate bone and the risk of tooth movement or loss (Jiang et al., 2024).

#### 2.3.3 Analysis of clinical application potential

As a viable therapeutic target, Piezo1 offers a promising avenue for refining OTM , though its full clinical utility requires further validation. Recent studies have shown that regulating the activity of the Piezo1 channel achieves precision calibration of the mobile mechanics of teeth. It is noteworthy that the inhibitive effect of drugs on the dental troughs slows the movement of teeth and inhibits rational bone absorption. This dual control mechanism provides a strategic framework for the protection of the structural integrity of the toothbone and toothbone tissues (Zheng et al., 2024).

Clinical Relevance of Drug-Based and Biomaterial Approaches in Modulating Piezo1,current state of knowledge has shown that the Piezo1 ion channel can be regulated by the use of pharmacological methods or biomaterial approaches to regulate periodontal tissue responses to mechanical stimuli, thereby affecting the dynamic mechanism of tooth movement (Pontes and Mendes, 2023). Localized delivery of Piezo1 agonists or antagonists can regulate periodontal tissue responses to mechanical stimuli and thus influence the dynamic process of tooth movement. The strategy has potential beyond increasing the effectiveness of orthodontic therapies and also reducing adverse impacts of an orthodontic therapy (Ayidağa and Kamiloğlu, 2021; Kono et al., 2025).

Treatments that focus on the root resorption might be improved by studying the role of Piezo1, which may subsequently be used in the future to treat the resorption of the root. There is one hypothesis that the inhibition of osteoclast activity through the inhibition of Piezo1 can suppress the root resorption (Qi et al., 2020). Although this is an exciting clinical intervention, there is a need to conduct more research to confirm its efficacy. Also, studies on Piezo1 can benefit the field of orthodontics by enhancing knowledge on the biological effects of pivoting teeth in addition to offering new prospects and interventions to improve the field.

### 2.4 Molecular pathways through which Piezo1 influences the quality of alveolar bone

#### 2.4.1 Regulation of osteogenesis-related factor expression

As a primary regulator of osteogenesis, RUNX2 is a vital factor involved in the regulation of osteoblast differentiation and activity. Such controlled activity also has a great influence on bone formation (Chiticaru and Ioniță, 2024). Piezo1 is a mechanosensitive ion channel that perceives the mechanical signal and translates it into intracellular signals, therefore, regulating the expression and activity of RUNX2. Piezo1 has also been reported to mediate alveolar bone remodeling during orthodontic tooth movement which is mediated by mechanical forces (Du and Yang, 2023). Additionally, Piezo1 activity mediates RUNX2 transcription via the Wnt/β-catenin signaling pathway, which subsequently promotes osteoblast activity and differentiation that subsequently triggers osteoblast activity and differentiation (Nirmala et al., 2020). RUNX2 also promotes downstream osteogenesis genes, including osteocalcin (OCN) and osteopontin (OPN) to boost bone formation and mineralization of the bone matrix (Du and Yang, 2023).

A number of exogenous stimuli such as microRNAs (miRNAs) and extracellular signaling molecules have been observed to control RUNX2 activation in osteoblast differentiation by controlling its inhibitory form (Wang et al., 2022). Piezo1 is therefore expected to play a role in controlling bone formation and alveolar bone remodelling during OTM by regulating the inhibitory form of RUNX2 transactivity.

The alkaline phosphatase (ALP) is a popular bone formation and osteoblast marker (Wang J. et al., 2023). Piezo1 stimulation improves the kinetics of ALP activation and fast signaling, which means that mechanical forces delivered through Piezo1 also stimulate osteoblast metabolism and collagen production. Moreover, osteoblastic stimulation of Piezo1 is associated with the stimulation of Alpl and collagen mineralization of the bone matrix, as well as with upregulation of the Wnt/β-catenin and BMP/Smad signaling pathways that play a critical role in regulating osteoblastic differentiation and activity (Iwata et al., 2023). The expression of the Alpl and collagen, and the mineralization of the bone matrix by Piezo1, is likely maintained by the activation of the Wnt/β-catenin and BMP/Smad signaling.

Piezo1 is a channel that mediates mechanical signals that result in elevation of intracellular calcium level in osteoblasts that is involved in bone formation (Du et al., 2024). Piezo1 is a mechanosensor, which regulates bone formation process in osteoblasts. This contributes to the idea that Piezo1-related signal transmission in osteoblasts depends on the rise of calcium and is facilitated by mechanic stimulation (Li et al., 2021). Signaling molecules of the mineralization process are released by mineralizing osteoblasts and include osteocalcin and osteopontin protein families. The expression of these genes is stimulated by activation of Piezo1, which leads to the development of a more mineralized matrix. Also, this signaling pathway can affect mineral deposition via strain-mediated processes that use CAMKII and ERK (Liu et al., 2025). Thus, the expression of Piezo1 is likely to have a variety of effects and impacts on osteoblast activity, albeit at varying degrees. The outcome of these studies implies that Piezo1 is a regulatory protein in mineralization particularly during abnormal conditions.

#### 2.4.2 Molecular foundations of bone remodeling equilibrium

Wnt/β-catenin signaling is a major regulator of bone remodeling and mineralization and Piezo1 is closely linked to the pathway (Yang et al., 2025). Being a mechanosensitive ion channel, Piezo1 may be triggered by mechanical stimulation which causes the Wnt/-catenin signaling to be initiated and consequently osteoblast differentiation and functioning. Moreover, Piezo1 activation has been reported to increase intracellular levels of β-catenin, an intermediate in the Wnt signal pathway (Tang et al., 2022). Osteoblastic activity and bone forming capacity is further promoted by the augmented availability of β-catenin.

Bone remodeling balance mainly relies on the accurate control of the Wnt/β-catenin pathway (Marian et al., 2024). Piezo1 is also involved in bone modeling by mediating osteoblast-osteocyte communication in bone modeling a process that is critical for alveolar bone remodeling. During OTM. Piezo1 maintains tissue homeostasis by mediating osteoblast-osteocyte communication, a process essential for skeletal homeostasis, which is a requisite in the skeletal homeostasis.

The bone morphogenetic proteins (BMPs) are also key regulators of the bone metabolism which is vital in formation of a vascularized and mineralized bone mass. In pathological conditions, the stiffening of the matrix may induce BMPIIa signaling mediated by Piezo1 that promotes differentiation and functional maturation of osteoblasts, which is supported by prior research (Sugimoto et al., 2017). Additionally, Piezo1 is widely expressed in osteoblasts, which highlights its significant role in the development of osteoblast-mediated bone remodeling.

Inflammatory factors play a complex role in bone remodeling, which can both promote bone resorption and influence bone formation. Under the regulation of Piezo1, the expression levels of inflammatory factors may affect the balance between osteoblasts and osteoclasts (Pantsulaia et al., 2023). This regulation is not limited to regulating the generation of broken bone cells, but has far-reaching effects on the dynamics of the musculoskeletal cells. For example, estrogens, as a key medium, are capable of fine-tuned cell reactions in micro-skeletal cell separation environments; and, more importantly, they tend to inhibit abnormal expressions of inflammatory factors (e.g. TNF-α and IL-1), thereby maintaining a balance conducive to bone formation. (Subarajan et al., 2024).

Piezo1 is an important regulatory factor for the response of cells to mechanical tissue damage, in the process the activation of Piezo1 will facilitate the release and activity of inflammation media. These media, in turn, destroy the balance of the rebranding. Therefore, the processes of bone remodeling during OTM and the maintenance of alveolar bone are important issues, a process that can be better understood by investigating how Piezo1 interacts with local inflammatory factors; this knowledge is critical for improving the efficacy of orthodontic treatment and clinical outcomes..

#### 2.4.3 Future directions for the development of therapeutic strategies

The role of Piezo1 in bone remodelling and maintenance of alveolar bone has been of growing interest. Consequently, there have been efforts to develop non-toxic small-molecule drugs by targeting Piezo1, effectively increasing osteogenesis and vascularization (Du and Yang, 2023). In a study, MCB-22-174 has been designed to activate Piezo1, which consequently promoted osteogenesis, and vascularization (Du and Yang, 2023).

In addition to medication, Piezo1 has become a powerful candidate for the genetic treatment of skeletal diseases. The idea of treating osteoporosis and other whole-body osteoporosis is presented through bit-specific regulation of Piezo1. Specifically, conditional regulation of this channel, with the precision of the CRISPR/Cas9 genome editing technology, provides an effective method of deconstructing the cytodynamic properties of the bone and the network of signals that regulate bone conversion. (Knothe and Springfield, 2005).

Beyond the theoretical framework, gene-based interventions have demonstrated significant potential for enhancing the structural integrity of the bones. This improvement was mainly due to the activation of the regenerative cascade response, which facilitated the cell-driven matrix synthesis and subsequent mineralization processes (Chen et al., 2025). Based on the above-mentioned findings, future research should focus on facilitating the transition of Piezo1 target-to-geotherapy from laboratory to clinical. The main objective must be a rigorous assessment of the stability and safety of long-term treatment in aerobic expansion into a complex biological environment. The establishment of these parameters is essential for the successful integration of genetic regulation techniques into clinical management of bone-related diseases.

With the increasing clarity of Piezo1’ s multiple functions in dental biology, its potential to optimize orthotic treatment at molecular levels has become a major research frontier. This mechanically sensitive channel, as a key regulatory factor for cellular reactions, determines the dynamic process of dental morphology, structural stability and tissue remodelling. The expression or activity of the Piezo1 regulatory factor provides a potential mechanism for the precise calibration of the rebranding results, which results from abnormal loads (Lin et al., 2022). The combination of Piezo1 testing with existing clinical and molecular prediction indicators marks a shift towards more individualized models. The application of these biomarkers has improved treatment efficiency and patient dependence, while protecting the quality of the tooth trough (Hijazi et al., 2024). Ultimately, maintaining the structural integrity of the toothbone is critical to maximizing the success of treatment and improving long-term patient anticipation. Continued exploration in this area will help to bridge the gap between basic mechanics biology and advanced clinical practice.

## 3 Conclusion

This overview is based on system combing that the Piezo1 ion channel plays an irreplaceable “mechanical-biological switch” role in the OTM . The available evidence provides ample evidence that Piezo1 not only captures the aberrational signals as the primary mechanical sensor in the dental membranes (PDL) cells, but also activates key signal axes such as YAP/TAZ, Wnt/beta-catenin and BMP/Smad by channeling the ca2+ internal flow, so that the balance between bone cells and broken bone cells is finely reconciled.Its unique “two - way regulation“ effect–i.e., the promotion of coordination between pressure - side bone absorption and tension - side bone formation–is the cytological cornerstone for efficient dental movement. .

The long - standing challenge in the treatment of malocclusion is to convert macromechanical loads into controlled biological responses. The Piezo1 discovery provides a molecular solution to this classic mechanics problem. By regulating key bone transformation genes (e. g. RUNX2, RANKL/OPG) through Piezo1, we not only confirm the introduction of “mechanical transferor theory“ in the introduction, but also shift the treatment of malformations from empirical force regulation to a new level of molecularly driven biological intervention.

From a scientific point of view, Piezo1 has demonstrated enormous clinical potential as a treatment target. Through the use of specific agonists (e.g. Yoda1 or MCB-22-174) or inhibitors (e.g. GsMTx4), clinicians are expected to achieve personalized “acceleration“ or “brakes“ of the pace of dental movement, while preventing complications such as the absorption of teeth by precision calibration of bone modification processes. Future research should focus on two directions: the development of a local drug delivery system targeting PDL or toothbrush to circumvent the full-body reaction that Piezo1 may bring to its wide distribution; and the use of genetic editing techniques such as CRISPR/Cas9 to dig deeper into the dynamic expression patterns of Piezo1 in a micro-inflammation environment.
