# Supplementary material for: Piezo1 ion channel: a core target for mechanotransduction in orthodontic alveolar bone remodeling
Source: Front Cell Dev Biol. 2026 May 8;14:1800626. doi: 10.3389/fcell.2026.1800626 (PMC13195014; doi:10.3389/fcell.2026.1800626)
Supplement: Supplementary file 1 [file DataSheet1.docx]

Supplementary Material

# Supplementary Figures and Tables
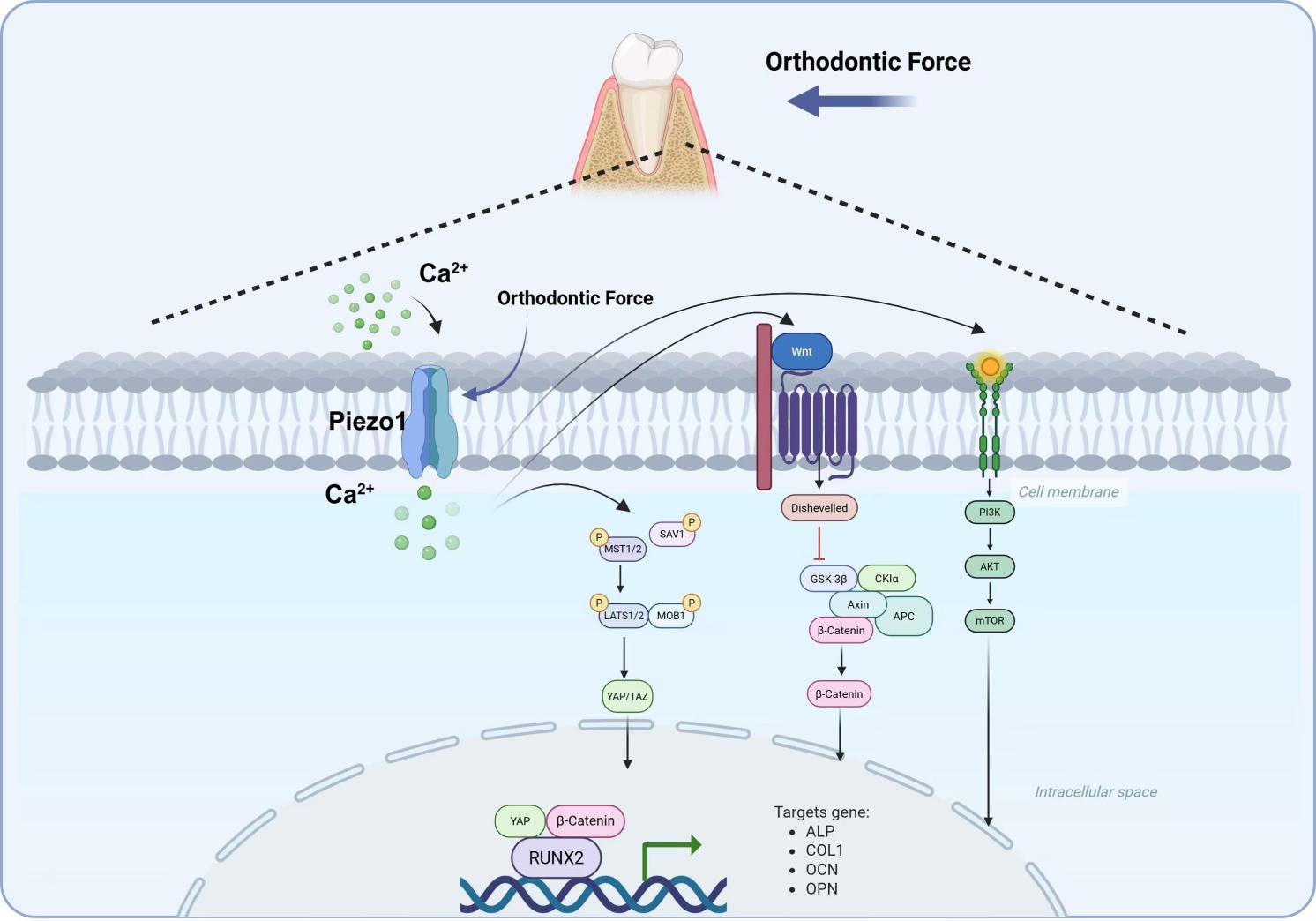


**Figure 1.**This figure illustrates the molecular mechanism by which the mechanosensitive ion channel Piezo1 regulates alveolar bone remodeling under orthodontic force through multiple signaling pathways, achieving the molecular network of adaptive alveolar bone remodeling under orthodontic force via multi-pathway interactive regulation of osteogenesis-related gene expression.


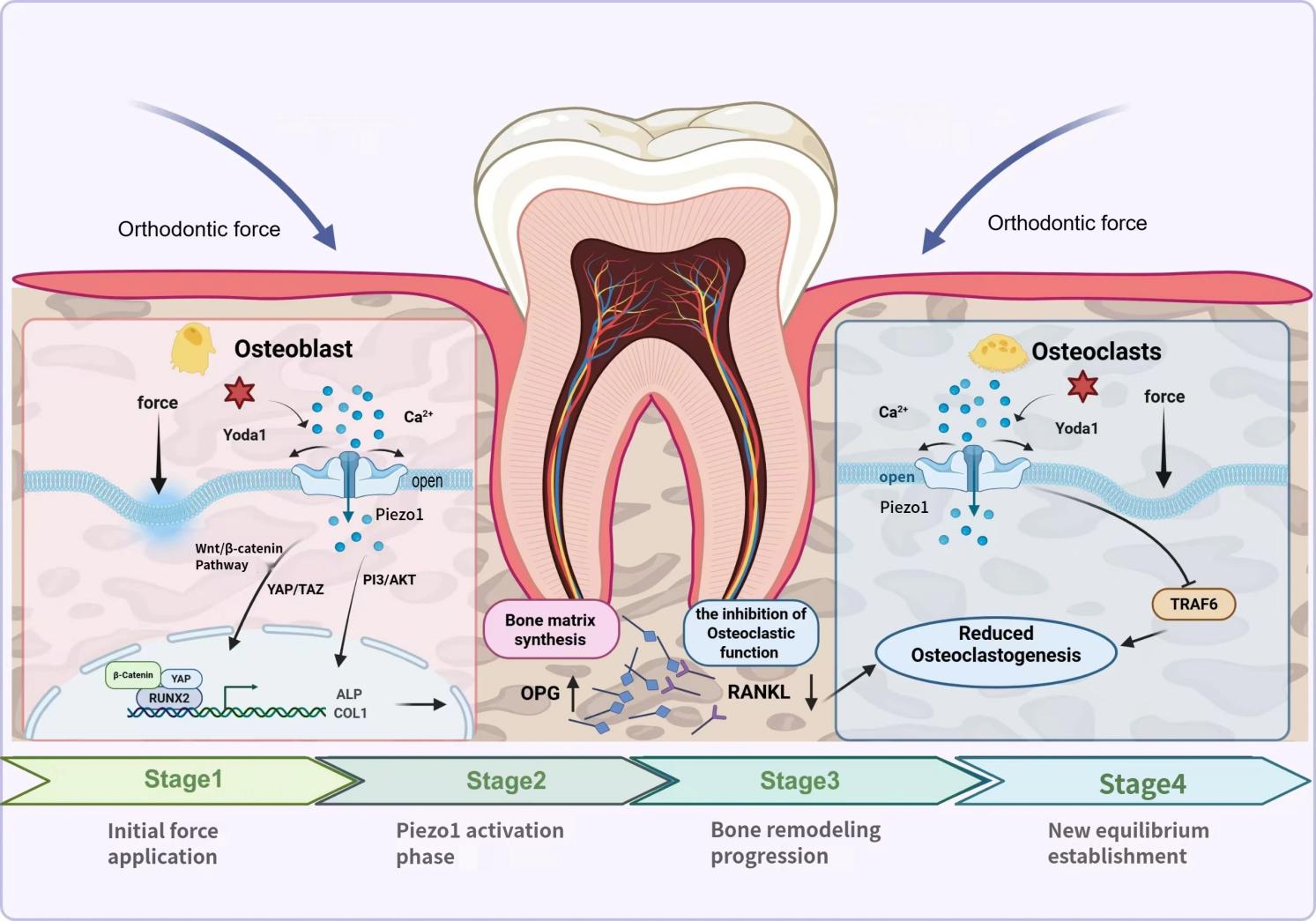


**Figure 2.**The figure illustrates the four-stage mechanism by which the Piezo1 mechanosensitive ion channel, under orthodontic force, regulates the signaling pathways of osteoblast-mediated bone formation and osteoclast-mediated bone resorption, thereby promoting alveolar bone remodeling and achieving a new equilibrium in tooth movement.
